# Prediction of Preeclampsia Using First-Trimester Uterine Artery Doppler and Pregnancy-Associated Plasma Protein-A (PAPP-A): A Prospective Study in Chhattisgarh, India

**DOI:** 10.7759/cureus.22026

**Published:** 2022-02-08

**Authors:** Esha Das, Vinita Singh, Sarita Agrawal, Saroj K Pati

**Affiliations:** 1 Obstetrics and Gynecology, All India Institute of Medical Sciences, Raipur, IND; 2 Radiodiagnosis, All India Institute of Medical Sciences, Raipur, IND

**Keywords:** hypertension in pregnancy, first trimester screening, uterine artery doppler, preeclampsia, papp-a

## Abstract

Introduction

Preeclampsia is a major contributor of maternal and perinatal morbidity and mortality. Uterine artery waveform and biomarkers like pregnancy-associated plasma protein-A (PAPP-A) may reflect the pathophysiology of preeclampsia. Thus, we aim to find out whether abnormal uterine artery pulsatility index (PI) and low serum PAPP-A in the first trimester can predict preeclampsia.

Methodology

Antenatal women at 11-13^+6^ weeks of gestation visiting All India Institute Of Medical Science (AIIMS) in Raipur were enrolled after informed consent. Uterine artery Doppler was done with the early anomaly scan at 11-13^+6^ weeks. Serum levels of PAPP-A were analyzed. The women were followed up at intervals up to delivery. Incidence of preeclampsia and gestational hypertension was noted.

Results

The incidence of preeclampsia was 12.7%, and that of gestational hypertension was 4.9%. The mean uterine artery PI among those who developed hypertension in pregnancy was 2.007, which was significantly higher than the unaffected group (p=0.01). The first-trimester uterine artery PI as a screening tool showed a sensitivity of 68%, specificity of 52.99%, and detection rate (DR) of 55.63%.

The mean PAPP-A MoM of the affected group was 0.67 which was significantly higher than the unaffected group (p<0.001). The first trimester PAPP-A as a screening tool showed a sensitivity of 28%, specificity of 90.6%, and DR of 79.58%.

Conclusion

Both the tests were concluded to be good predictors of hypertension in pregnancy. Identification of high-risk factors, screening, and surveillance are of utmost importance in order to predict preeclampsia and initiation of preventive therapy.

## Introduction

Preeclampsia (PE) is a pregnancy-specific syndrome that is the second single leading cause of maternal mortality. It accounts for 14% of maternal deaths worldwide and 29.54% of maternal deaths in India [1, 2]. It is contributory to maternal and perinatal morbidity, and it is devastating and life-threatening for both mother and the baby [3]. Ten percent of women develop high blood pressure during pregnancy, 2% to 8% develop complications. Preeclampsia is a multisystem disorder that involves the hepatobiliary, renal, neurological, and hematological systems [4].

Preeclampsia results from reduced invasion of trophoblastic cells into the myometrial portions of the spiral arteries, leading to increased resistance to flow in the uteroplacental unit, which is transmitted upstream to the uterine arteries [5]. This results in an increased pulsatility index (PI). Thus, uterine artery Doppler waveform analysis has the potential to predict pregnancy complications associated with uteroplacental insufficiency before the onset of clinical features [6].

Placental products are released in the bloodstream during the formation of the placenta. They are the biochemical markers that reflect the pathophysiology of defective placentation. Pregnancy-associated plasma protein-A (PAPP-A) is a syncytiotrophoblast-derived protease for insulin-like growth factor binding protein-4 (IGFBP-4). Low PAPP-A levels result in low circulating levels of free insulin-like growth factor (IGF), which may cause preeclampsia and fetal growth restriction. There is recent evidence that low first-trimester maternal serum PAPP-A in chromosomally normal pregnancies is associated with an increased risk for subsequent development of preeclampsia [7, 8].

Early prediction of preeclampsia is still a challenge. Several markers have been studied by researchers in the past. Based on the literature review PAPP-A and uterine artery Doppler in the first trimester have shown promising results in the prediction of preeclampsia. However, most of the studies available were conducted on the Caucasian and African-American populations. Data from the South Asian population is still insufficient [3].

Our objective is to find out whether abnormal uterine artery Doppler and/or maternal PAPP-A levels at 11 to 13+6 weeks can predict preeclampsia.

Previously, second-trimester uterine artery Doppler examination was used as a screening tool for preeclampsia in many studies. But trophoblast invasion is maximal in the first trimester. Thus there is justification in the evaluation of uterine artery Doppler in the first trimester of pregnancy.

As per the institute protocol, at 11-13^+6^ weeks, routine screening for aneuploidy is done by early anomaly scan and dual markers, which includes PAPP-A and free beta human chorionic gonadotrophin (βhCG). Thus it will not incur additional costs to the patient. This has to be kept in mind, especially in a developing country like India [8].

## Materials and methods

This was a prospective analytical study done in the All India Institute of Medical Sciences (AIIMS) in Raipur after getting approval from the Institute Ethics Committee. The study was conducted from September 2018 to March 2020 over a period of one and a half years. The sample size was calculated by Cochran's formula [9]. The prevalence of preeclampsia was taken as 10% [10]; thus, the sample size was calculated to be 138. The study included all antenatal women attending AIIMS out-patient department (OPD) at or before 11-13^+6^ weeks gestation, willing to follow-up and give consent for the study. All the women were residents of the Chhattisgarh state. The study excluded women with multifetal gestation, congenital anomalies in the fetus, concurrent medical illness (like hypertension, overt diabetes mellitus, cardiac, renal, hepatobiliary, hematological and neurological disorders), and ones on antihypertensive medication. After taking informed consent for participation in the study, detailed history, a general and systemic examination was done at the first visit. Particulars like age, parity, body mass index (BMI), education, socioeconomic status (modified BG Prasad Scale 2018 [11]), occupation, religion, residence, and family history of hypertension were noted. After confirming dates and fetal cardiac activity, antenatal women were advised to undergo uterine artery Doppler along with the routine nuchal translucency (NT)/ nasal bone (NB) scan at 11-13^+6^ weeks. To reduce inter-observer discrepancy, all the scans were done in the Department of Radiodiagnosis at the AIIMS in Raipur by radiologists with more than six years of experience using the GE LOGIQ S8 machine (GE Healthcare, Chicago, Illinois). The uterine artery pulsatility index (UtA-PI) was noted. The values of PAPP-A in multiples of median (MoM) were noted from the dual markers report. Dual markers test was done in the Department of Biochemistry at the AIIMS in Raipur.

The study population was divided into four groups; the first one being patients with abnormal first-trimester uterine artery Doppler and normal PAPP-A, the second one being patients with abnormal PAPP-A and normal first-trimester uterine artery Doppler, the third one where both investigation results were abnormal, and last those where both investigation results were normal.

Follow-up visits were as follows: once every four weeks until 28 weeks gestation, once every 2 weeks until week 36, and weekly until week 40 or until delivery. In each follow-up visit, the blood pressure and urine dipstick was noted. Diagnosis and definition of hypertensive disorders of pregnancy (preeclampsia and gestational hypertension) were made as per the American College of Obstetricians and Gynecologists (ACOG) 2013 criteria [10].

Statistical analyses were performed using a Statistical Package for Social Sciences software (SPSS) version 16 (IBM, Armonk, New York). Student t-test (independent t-test) was applied to compare the PAPP-A MoM and UtA-PI between the normotensive and those who developed hypertensive disorders.

The Chi-square test was applied to see the association between outcome and demographic characteristics. The Receiver Operating Curve (ROC) was constructed for PAPP-A MoM and UtA-PI values.

For all statistical analyses, p<0.05 was considered significant, and p<0.001 was considered highly significant.

## Results

A total of 540 antenatal women at or before 11-13^+6^ weeks presented to the out-patient department during the study period. Of those, 162 were excluded as they had one or more of the exclusion criteria. Amongst the remaining women, 98 presented with outside reports of early anomaly scan without a uterine artery Doppler, and 134 women were not willing to consent for follow-up; hence they were excluded. Out of a total of 146 women who consented for the study, four were lost to follow-up and were excluded during the analysis of data, and thus a total of 142 women’s data were subjected for the final analysis.

As described in Table 1, 38.8% of the women were 26-30 years of age, 2.8% of women were 18-20 years of age, 2.8% of the women were 36-40 years of age. The youngest woman was 19 years of age, and the oldest woman was 38 years of age. The study population comprised 65.5% of primiparous women and 34.5% of multiparous women. Amongst all the women, 35.9% of the women had higher secondary education, which was the majority, while 2.1% of the women had primary education. Most of the women were homemakers (75.3%) while few of them (24.7%) employed. Among the total women, 79.6% belonged to Class I socioeconomic scale while none of the women belonged to Class IV and Class V. Majority of the women were urban dwellers (93.0%) while very few (7.0%) of the women lived in rural areas. The majority of the women were Hindus (90.4%), followed by Muslims (5.6%), Christians (2.8%), and Sikhs (0.8%). In the study population, 7% of the women had a family history of hypertension, while 93% did not.

**Table 1:** Demographic characteristics of the study population

Demographic characteristics		Number of women (n=142)	Percentage
Maternal age	18-20	4	2,8
	21-25	48	33.8
	26-30	55	33.8
	31-35	31	21.8
	36-40	4	2.8
Parity	Primiparous	93	65.5
	Multiparous	49	34.5
BMI	<18.5	4	2.8
	18.5-24.9	61	43.0
	25-29.9	62	43.7
	≥30	15	10.5
Education	Post Graduate	25	17.6
	Graduate	46	32.4
	Higher Secondary	51	35.9
	Secondary	17	12.0
	Primary	3	2.1
Socioeconomic class (modified BG Prasad Scale 2018)	I	113	79.6
	II	28	19.7
	III	01	0.7
Occupation	Housewife	107	75.3
	Working	35	24.7
Religion	Hindu	129	90.8
	Muslim	8	5.6
	Christian	4	2.8
	Sikh	1	0.8
Area of residence	Rural	10	7.0
	Urban	132	93.0
Family history of hypertension	Present	10	7.0
	Absent	132	93.0

As per Table 2, the incidence of preeclampsia was 12.7%, and the incidence of gestational hypertension was 4.9%. The incidence of hypertensive disorders in pregnancy (preeclampsia and gestational hypertension) was 17.6%. 

**Table 2:** Pregnancy outcome of the study population

Outcome	Number of women (n=142)	Percentage (%)
Normotensive	117	82.4
Preeclampsia	18	12.7
Gestational Hypertension	7	4.9

The mean of the first-trimester uterine artery PI values of those developing hypertensive disorders of pregnancy (2.00) is significantly higher (p=0.01) than the mean uterine PI of those who did not (1.51), as shown in Table 3

**Table 3:** Comparison of mean uterine artery PI (first trimester) among hypertensive disorders of pregnancy and normotensive PI: pulsatility index, SD: standard deviation

Normotensive		Hypertensive disorders of pregnancy		t-value	p-value
Mean uterine artery PI	SD	Mean uterine artery PI	SD		
1.51	0.56	2.00	0.86	-2.73	0.01

**Figure 1:**
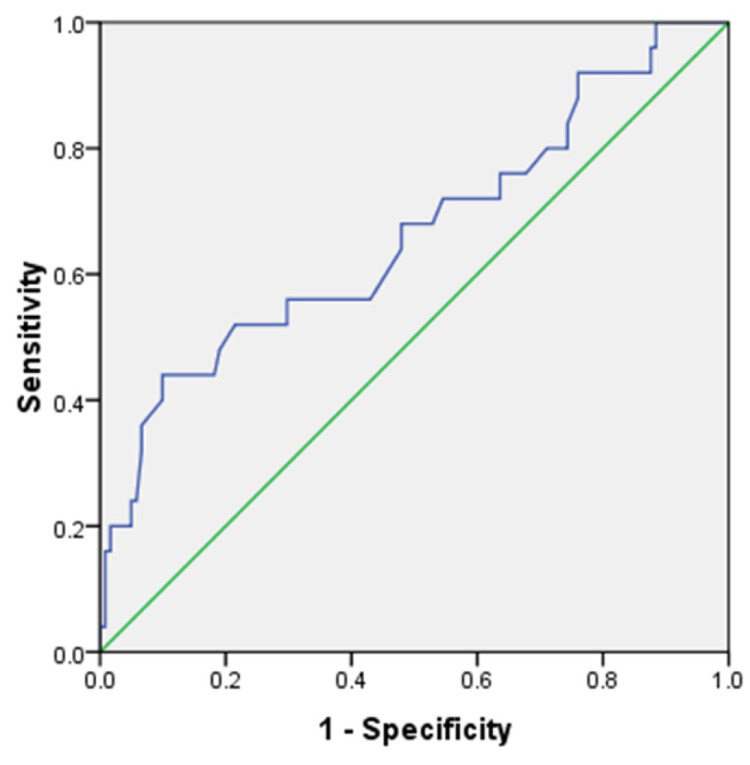
Receiver operator characteristic (ROC) curve of uterine artery pulsatility index

From the receiver operator characteristic curve of uterine artery pulsatility index (Figure 1), the area under the curve was 0.664. The best cut-off that maximized sensitivity and specificity was 1.48. The first-trimester uterine artery PI as a screening tool had a sensitivity of 68%, specificity of 52.99%, positive predictive value (PPV) of 23.61%, negative predictive value (NPV) of 88.57%, and a detection rate (DR) of 55.63%.

The mean first trimester PAPP-A MoM values of those who developed hypertensive disorders of pregnancy (0.67) were significantly lower than the mean PAPP-A MoM of those who did not (1.21, p<0.001) (Table 4).

**Table 4:** Comparison of mean PAPP-A MoM levels among hypertensive disorders of pregnancy and normotensive MoM: Multiples of Median, SD: Standard Deviation, PAPP-A: pregnancy-associated plasma protein-A

Normotensive		Hypertensive disorders of pregnancy		t-value	p-value
Mean PAPP-A MoM	SD	Mean PAPP-A MoM	SD		
1.21	0.71	0.67	0.39	-3.22	<0.001

**Figure 2:**
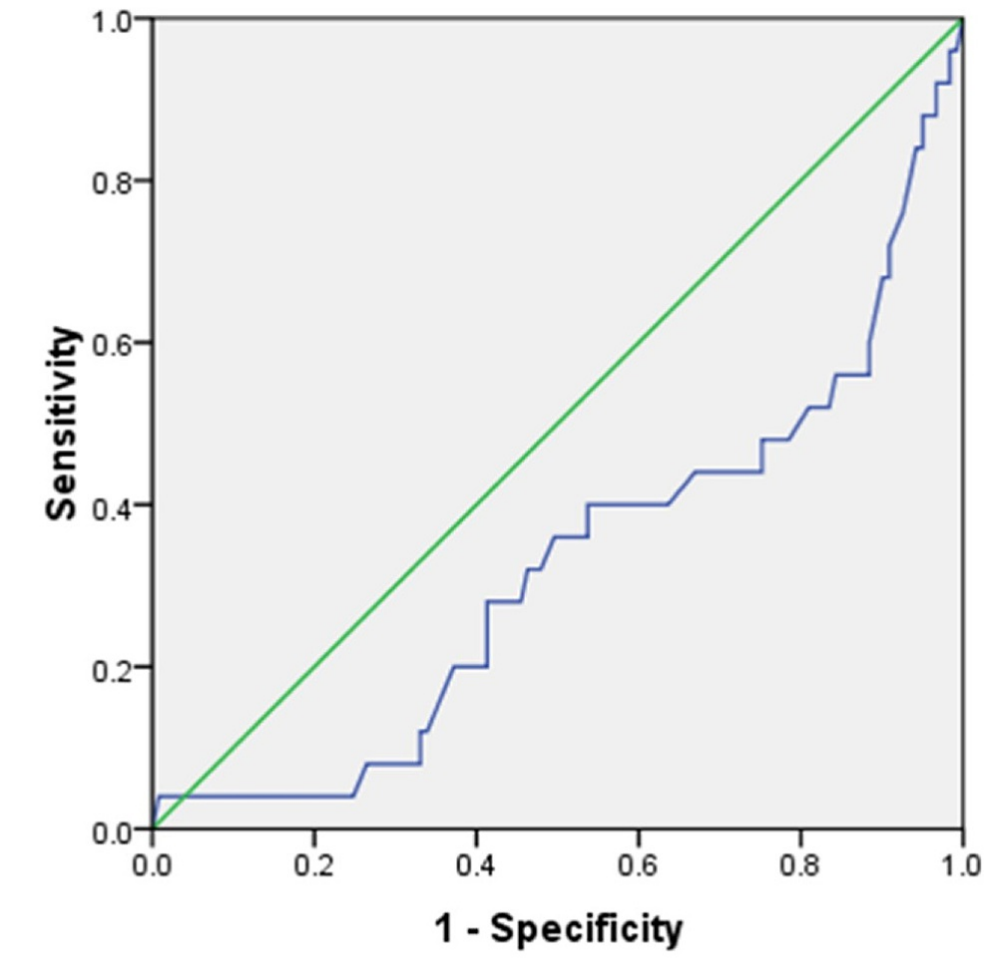
Receiver operator characteristic (ROC) curve for PAPP-A MoM MoM: Multiples of Median, PAPP-A: pregnancy-associated plasma protein-A

As per the receiver operator characteristic (ROC) curve for PAPP-A MoM (Figure 2), the area under the curve was 0.319. The best cut-off that maximized sensitivity and specificity was 0.41. The first trimester PAPP-A as a screening tool had a sensitivity of 28%, specificity of 90.6%, PPV of 38.89%, NPV of 85.48%, and a DR of 79.58%.

The incidence of preeclampsia was 66.7% when both the screening parameters were abnormal. The incidence of preeclampsia was 5.9% when both parameters were normal. When combined, their association with preeclampsia was highly significant (p<0.001) (Table 5).

**Table 5:** Comparison of hypertensive disorders of pregnancy among the study groups UtA-PI: uterine artery pulsatility index, PAPP-A: pregnancy-associated plasma protein-A

Screen characteristic	Hypertensive disorders of pregnancy		Normotensive n (%)	Total	χ^2^	Degree of freedom	p-value
	Preeclampsia n (%)	Gestational hypertension n (%)					
Both abnormal	4 (66.7%)	0 (0%)	2 (33.3%)	6	35.4	6	<0.001
UtA-PI abnormal, PAPP-A normal	5 (15.6%)	4 (12.5%)	23 (71.9%)	32			
UtA-PI normal, PAPP-A abnormal	4 (21.0%)	3 (15.9%)	12 (63.1%)	19			
Both Normal	5 (5.9%)	0 (0%)	80 (94.1%)	85			
Total	18	7	117	142			

## Discussion

Prediction of preeclampsia is an important strategy in order to prevent maternal and fetal morbidity and mortality associated with late diagnosis and inadequate management. Many studies are available where researchers have used different methods to predict preeclampsia. It was only until the 1980s that serum levels of biomarkers were studied to predict preeclampsia. In the present study, the efficacy of uterine artery Doppler and maternal serum PAPP-A in the first trimester as a screening tool for preeclampsia was studied. The incidence of preeclampsia was found to be 12.7%, and that of gestational hypertension was 4.9%; thus, the incidence of hypertension in pregnancy (preeclampsia and gestational hypertension) was 17.6% (Table 2). The incidence of preeclampsia in the aforementioned study groups was as follows: first group: 66.7%, second group: 15.6%, third group: 21.0%, and the fourth group: 5.9% (Table 5). Thus when both investigation results were abnormal, the incidence of preeclampsia was significantly higher (p<0.001).

The mean uterine artery PI value among those who developed hypertension in pregnancy (preeclampsia and gestational hypertension) was 2.007, which was significantly higher than the unaffected group (p=0.01) (Table 3). Satish et al. [12], G’omez et al. [13], and Narang et al. [7] also had a similar mean PI among the affected group: 2.34, 2.04, and 1.94, respectively.

Goetzinger et al. [14] did not find any significant difference in uterine artery PI values between affected and unaffected groups.

Uterine artery PI at 11-13^+6^ weeks was considered a good predictor for hypertensive disorders of pregnancy with a sensitivity of 68% and specificity of 52.99% at a cut-off of 1.48 (as obtained from the ROC curve, Figure 1). A similar cut-off (1.52) was also obtained in a study by Staboulidou et al. [15]. Narang et al. [7] conducted a study in Uttar Pradesh, India, and showed similar sensitivity (75.9%) and specificity (79.6%). Similar sensitivity (64%) was also shown by Odibo et al. [16].

At the 95%th percentile of uterine artery PI, Martin et al. [17] and G’omez et al. [13] showed lower sensitivity (27% and 23.9%, respectively). Goetzinger et al. [14] also showed a lower sensitivity of 52%.

The PPV was 23.61%, and NPV was 88.57% for uterine artery PI in the screening for preeclampsia, according to this study. Singh et al. [18] conducted a similar study in India, and while the NPV was comparable (97.33%), the PPV was much higher (92%).

In this study, the mean PAPP-A MoM of the affected group (preeclampsia and gestational hypertension) was 0.67 (Table 4), which was comparable to the studies of Goetzinger et al. [14] and Spencer et al. [19] with mean PAPP-A MoM of 0.88 and 0.772.

From the ROC curve (Figure 2), the cut-off value for PAPP-A MoM at 11-13^+6^ weeks was 0.41 with a sensitivity of 28% and specificity of 90.6% in this study. Zhong et al. [20] showed similar sensitivity and specificity of 16% and 93%, respectively.

Odibo et al. [16] showed a higher sensitivity of 58% for PAPP-A. Staboulidou et al. [15] had found a cut-off value of PAPP-A MoM at 0.58 for preeclampsia. Patil et al. [21] showed a PPV of 52% compared to 38.89% of our study at a cut-off of 0.5.

As shown in Table 5, the incidence of preeclampsia is 66.7% when both the screening parameters are abnormal. The incidence of preeclampsia becomes respectively in the affected group - 5.9% when both the parameters are normal. When combined, their association with preeclampsia is highly significant (p<0.001). Similar observations were made by Staboulidou et al. [15] and Satish et al. [12]. However, according to Odibo et al. [16], the median uterine artery MoM was higher in the group with early preeclampsia; it was not statistically significant. The PAPP-A MoM was significantly lower in the affected group. Thus a combination of all the parameters did not improve the screening capacity.

Poon et al. [22] found PAPP-A to be a good predictor of early-onset preeclampsia. There was no significant improvement by the addition of PAPP-A to the combination of maternal factors, mean arterial pressure (MAP), and uterine artery in the prediction of late PE and gestational hypertension. Goetzinger et al. [14] demonstrated that at 11-14 weeks gestation, ADAM12, PAPP-A, and uterine artery Doppler in combination with maternal characteristics identified 50%, 48%, and 52% of patients who developed preeclampsia, respectively. Thus a combination of all the parameters did not improve the predictability.

Tan et al. [23] published that UtA-PI, MAP, and placental growth factor (PlGF) predicted 90% of early-PE, 75% of preterm-PE, and 41% of term-PE, however, PAPP-A did not improve the performance of screening. Similarly, Narang et al. [7] also did not find the addition of PAPP-A to help in the prediction.

The limitation of the study was that some demographic characteristics of the study population were skewed. This was probably due to the study being set in a tertiary care center. Increased study duration with a greater sample size involving two or more centers located in different areas may have given better results. 

## Conclusions

Preeclampsia is a life-threatening disorder of pregnancy. Thus early detection of the disease at a low cost is of utmost importance. Uterine artery Doppler in the first trimester done along with early anomaly scan at 11-13^+6^ weeks has been found to be a good predictor of hypertension in pregnancy with moderate sensitivity and specificity. It is convenient and cost-effective to use PAPP-A as a biomarker since PAPP-A levels are routinely checked as a part of aneuploidy screening. It also has a high specificity for preeclampsia screening. It does not incur an additional financial burden to the patient. Thus PAPP-A can be considered a good screening tool for the detection of hypertension in pregnancy.

Identification of high-risk factors, screening, and surveillance are of utmost importance in order to predict preeclampsia. Preventive therapy, if initiated early on the basis of these screening tests, will help save the antenatal woman from the complications of preeclampsia.
